# Untold Stories of Future Engineers: Engineering Students’ Reflections in a Fiction-Based Ethics Course

**DOI:** 10.1007/s11948-026-00615-x

**Published:** 2026-08-03

**Authors:** Sara M. Andersson, Eva M. Brodin

**Affiliations:** 1 Department of Educational Sciences, Lund University, Box 117, Lund, SE-221 00 Sweden; 2 Division for Higher Education Development (AHU), Department of Educational Sciences, Lund University, Lund, Sweden; 3 Centre for Higher and Adult Education, Stellenbosch University, Stellenbosch, South Africa

**Keywords:** Ethics, Fiction, Higher education, Professional skills, Social responsibility

## Abstract

The integration of micro- and macroethical perspectives remains a challenge in engineering ethics education (EEE). Fiction-based EEE has been advocated as a pedagogical approach that can help students in this regard. However, how students actually use fiction to engage with micro- and macroethics remains underexplored. Therefore, this case study aimed to (1) identify how students’ engagement with fictions connected to micro and macro perspectives on engineering and technology ethics, (2) examine how students realized pedagogical potentials of fiction-based pedagogy in this regard, and (3) explore how the outcomes related to the pedagogical framing and activities of the course. The selected case was an elective, fiction-based EEE course, during which the written course work of students, teacher and student interviews, course materials, and data from workshop observations were collected for qualitative analysis. Findings show that several expected pedagogical potentials of fiction-based pedagogy were realized as students reflected on what guides human behavior and on the societal impact of technology, both within and beyond the fictions. However, students’ reflections rarely connected to their future professional role. These findings could be related to characteristics of the employed pedagogy and fictions, but also to students’ prior knowledge and expectations regarding professional engineering practice. We conclude that students may need targeted support towards connecting fictions with self and profession, and that fiction-based EEE may benefit from recognizing and responding to students’ pre-existing expectations on social responsibility and ethical agency in engineering.

## Introduction

Engineers contribute to the creation of physical environments and mediating tools that shape billions of human and non-human lives around the world. Simultaneously, they are themselves ‘also particularly situated actors, whose educational opportunities and work settings place constraints on what they learn, know, and do’ (Smith, 2021, p. 13). These circumstances create a compelling need to equip engineering students for complex ethical thinking in their future profession, preparing them to grasp how political, economic, and institutional meta-structures influence the ethical behaviors of engineers, individually and collectively – and vice versa (Byrne et al., 2022; Conlon, 2022; Monteiro & Sousa, 2024; Nguyen et al., 2020; Tormey et al., 2015).

In Sweden it is nationally regulated that engineering students should learn to make ethical judgements and ‘show insight into the role of technology … in society and individuals’ responsibility for how it is used, including social and economic aspects as well as environmental and work environment aspects’ (Higher Education Ordinance, 1993, p. 100, Annex 2), and similar criteria are maintained by several international accreditation boards (Hitt et al., 2023). However, making this connection between microethical perspectives (focusing on individual actors) and macroethical perspectives (embracing the societal level) has turned out to be a challenge for engineering students, and some of the most common pedagogical strategies in engineering ethics education (EEE) fall short in supporting students in this regard (Herkert, 2005; McAninch, 2023; Schiff et al., 2021).

A range of teaching strategies have been implemented in EEE to address perceived pedagogical needs, including e.g., case studies, group or class discussions, reviewing codes and rules, developing heuristics for ethical decision-making, team projects, service learning, communication with professional engineers, individual written assignments, literature study, games, and role play (Chance et al., 2025; Hess & Fore, 2018; Shen & Li, 2021). Like some of these, fiction-based pedagogy has been promoted as a promising approach to decrease the gap between microethical versus macroethical perspectives in students’ learning (Berne & Schummer, 2005; Burton et al., 2015, 2018; Hitt & Lennerfors, 2022; Hansen, 2018, 2021; Manià et al., 2018). However, empirical research on this matter in EEE is still scarce.

Hence, the overall purpose of this study was to explore how engineering students at the bachelor’s and master’s levels engaged with fictional texts in an EEE course led by a literary scholar at a Swedish university. The specific aims were to (1) identify how the students’ engagement with fictions connected to micro and macro perspectives on engineering and technology ethics, (2) examine how students realized pedagogical potentials of fiction-based pedagogy in this regard, and (3) explore how the outcomes related to the pedagogical framing and activities of the course.

### Engineering Ethics Education

The objectives of EEE, its content, teaching methods, and integration into engineering education, have been the subject of extensive debate (Berne & Schummer, 2005; Didier, 2000; Hess & Fore, 2018; Lennerfors, 2025; Li & Fu, 2012; Martin et al., 2021; Nguyen et al., 2020; Thoo & Strong, 2020; Walling, 2015). Part of this debate concerns the perceived deficiencies of the ‘dominant’ approach (Conlon, 2022), i.e. EEE pedagogy that focuses on the moral responsibilities of individual engineers and employs a normative, analytical approach to the topic (Hess & Fore, 2018; Mitcham, 2009; Smith, 2021). This approach has been criticized for treating ethics as a cognitive exercise disconnected from the individual’s moral sensibilities (Walling, 2015), failing to recognize that ethical decision-making in engineering is often a collective and complex process (Bucciarelli, 2008; Nguyen et al., 2020), excluding the action-dimension of taking responsibility in practice (Lennerfors, 2025), and disregarding societal (Martin & Polmear, 2022; Xavier et al., 2022) and macro-professional (Herkert, 2005) perspectives.

Conlon (2022) critically points out that viewing microethics in detachment from the social structures that condition the decision-making of individuals can result in unrealistic, burdening moralism. On the other hand, macroethical perspectives also need to be balanced, since the impact of social structures in detachment from individual agency can lead to a defeatist and complacent view of engineers as ‘dopes who merely manifest the demands of society … in their actions’ (Conlon, 2022, p. 228). Accordingly, the ‘micro-macro divide’ (Rottmann & Reeve, 2020) is a central critique of the current pedagogical landscape in EEE.

#### The Micro-Macro Divide and its Pedagogical Implications

The micro-macro framework (Herkert, 2005; McAninch, 2023) has been useful in exploring diversity in EEE practice and learning goals (e.g. Martin et al., 2021; Polmear et al., 2019), but also in conceptualizing tensions and gaps within EEE. For instance, Schiff et al. (2021) found that engineering students struggled with connecting their macroethical awareness to their own disciplines and careers, because they perceived the engineering profession to deal with technical matters only. This perception was reinforced by the separation of social and technical aspects of engineering, i.e. a socio-technical divide, in curriculum and program design (Schiff et al., 2021). Moreover, they found that students could draw on ethical values from their personal lives to inform their sense of micro-professional ethics, while their understanding of macroethical issues tended to be detached from this experiential basis (ibid.).

Several pedagogical strategies for bridging the micro-macro divide in EEE have been proposed. Rottmann and Reeve (2020) suggest guided case analyses in which teachers support students’ reasoning as they work towards an integrated understanding of micro- and macroethical perspectives. Schiff et al. (2021) recommend integrating ethics across the curriculum through disciplinary approaches to macroethical issues. Martin et al. (2023) highlight real-life, interdisciplinary projects conducted in collaboration with external stakeholders, such as NGOs or local governments. Herkert (2005) promotes the integration of methods and concepts from the field of Science, Technology and Society (STS) into EEE, and Herkert et al. (2009) outline how such integration can be achieved, e.g. through laboratory sessions where students can combine ethical reflection with practical work in a familiar environment. Nonetheless, fiction-based pedagogy is still another approach to be elaborated upon in this article.

### Fiction-Based Engineering Ethics Education

In the present article, fiction-based EEE is taken to mean ethics education for engineering students in which narrative art such as short stories, novels, plays, or filmic fiction plays a significant part. As such, fiction-based EEE can provide a platform for interdisciplinary approaches to teaching and learning (Hansen, 2018; Odle & Bassett, 2021; Summet & Bates, 2020).

#### Interdisciplinary Approaches to EEE

EEE can be viewed as an inherently interdisciplinary practice, where e.g. philosophy, sociology, critical theory, organizational studies, law, and moral and social psychology are connected to engineering design (Tormey, 2025). Interdisciplinary approaches allow engineering students to develop complex understandings of the paradigms, assumptions, and institutional contexts that shape technologies, engineering education, and engineering practice: what they are, and how they are changing (Christensen et al., 2022; Tormey, 2025). Thereby students can become ‘whole’ engineers who are technically skilled, globally oriented, and socially responsible (Hitt et al., 2023, p. 457), as well as competent collaborators who can address critical problems facing mankind (Byrne et al., 2022).

Fiction-based EEE shares in both the affordances and challenges of interdisciplinary education. It is often taught by faculty from the Social Sciences or Humanities, who tend to rely on their own disciplinary understanding in their teaching (Xavier et al., 2022). Consequently, students may lack sufficient support to see how ethics are relevant to their work as engineers (Hansen, 2018; Jarzemsky et al., 2023; Martin & Polmear, 2022; Xavier et al., 2022; York, 2018).

#### Teaching Ethics Through Narrative art

The use of literature to teach ethics spans the scholarship and educational traditions of many cultures and can be traced back to the earliest works on the purposes of poetry and stories (Hogan, 2022). Through e.g. the Bildung tradition and liberal arts education, the practice has played a longstanding part in modern higher education (Berne & Schummer, 2005). Within the context of EEE, the pedagogical use of narrative art has been discussed for some decades as an approach that allows students to shift between and combine individual, professional, and societal perspectives as they engage with ethical dilemmas represented in literature or film (Berne & Schummer, 2005; Burton et al., 2015, 2018; Hansen, 2018, 2021; Hitt & Lennerfors, 2022; Jalali et al., 2021; Manià et al., 2018; Sleezer & Bates, 2020).

Suggested ways of using literary and filmic fictions in the EEE classroom include individual reading/viewing followed by facilitated group discussions and/or written reflections (e.g. Berne & Schummer, 2005; Hansen, 2018; Hitt & Lennerfors, 2022; Jalali et al., 2021; Sleezer & Bates, 2020; Summet & Bates, 2020), scaffolded readings/viewings in which students’ attention is guided to certain aspects of form and content (e.g. Burton et al., 2023; Odle & Bassett, 2021; Hansen, 2021), theory-informed analysis of ethically significant situations in fictions (e.g. Burton et al., 2018), and as inspiration for creative writing or collaborative projects (e.g. Manià et al., 2018; Schroeder, 2020).

#### Four Pedagogical Potentials of Fiction-Based EEE

Fiction-based EEE is commonly related to four pedagogical potentials, which arise from the properties of narrative art (Berne & Schummer, 2005; Burton et al., 2018; Hansen, 2018, 2021; Jalali et al., 2021; Schroeder, 2020). These are:

(i) *Application of theoretical approaches*. Fictions provide a rich material that allows for multiple interpretations. Students can apply various theoretical lenses to fictional ethical issues (Burton et al., 2018; Schroeder, 2020; Hansen, 2018, 2021), and students’ activities can also be designed to strengthen particular analyses, for instance, by repeating certain questions in relation to multiple fictions (Hansen, 2021).
(ii) *Altering positions.* Fictions allow students to develop their empathetic ability through identification with and immersion in characters (Burton et al., 2015; Hitt & Lennerfors, 2022; Jalali et al., 2021; Manià et al., 2018; Sleezer & Bates, 2020). It has been argued that fully taking the perspective of a fictional character entails both cognitive and affective immersion (Jalali et al., 2021), which can create a distance to accustomed positions that helps students to reflect upon their own frames of reference and subject positions (Hitt & Lennerfors, 2022; Sleezer & Bates, 2020), on the one hand, and to detect potential social inequity within existing paradigms (Manià et al., 2018), on the other hand.
(iii) *Consideration of context.* Given that engineers’ decision-making depends on several contextual factors in practice (Hitt & Lennerfors, 2022), and that fictional texts supply rich material for stakeholder identification and analysis (Summet & Bates, 2020), students can realize that their risk perception is not only subjective, but it is also socially embedded and thus vulnerable to uncritical acceptance of (un)ethical practices (Berne & Schummer, 2005).
(iv) *Imagining possible futures.* Engineering students need to be prepared for future work with technologies that do not yet exist (Berne & Schummer, 2005), and fiction is a low-risk resource for testing new ideas (Hansen, 2021). Furthermore, fiction can provide insight into hopes and fears that inform public understandings and opinions regarding emerging technologies, which are important to consider in EEE even if they are far removed from reality (Berne & Schummer, 2005; Sleezer & Bates, 2020). Finally, access to possible futures through fiction can have an emancipatory value as it shows students alternatives to the current reality and thus the possibility for changing the status quo of society (Hansen, 2021).

These pedagogical potentials all have a bearing on micro-macro integration. As far as application of theoretical approaches goes, the micro-macro lens itself, various critical frameworks, or literary devices such as allegory can be applied to fictions to promote awareness of the connections between individual realities and actions and overarching social structures. Altering positions may enable seeing and juxtaposing individual perspectives in a bigger social picture. Finally, consideration of how individual actions are conditioned by (social) context and imagining of possible (societal) futures sparked by individual actions have obvious relevance for the exploration of interplay between micro- and macroethical perspectives.

## Methodology

### Single Case Study

The study was based on a single case design and followed Merriam’s (1998) approach to conducting case studies in education. Merriam (1998) argues that a case is, through its situatedness in a real-life situation, naturally delimited in terms of time, space, and/or involved actors. Based on this definition, our case was delimited to the teacher and students in the studied fiction-based EEE course. Multiple case studies are typically considered preferable to generate solid results (Gerring, 2004; Merriam, 1998), but a single social setting enables more nuanced understanding of the entire context (Dyer & Wilkins, 1991) which was needed for the purpose of our study.

The case was selected for the teacher’s explicit pedagogical intentions (further developed in section  “Making Students ‘Round Themselves out’”) to help students embrace both micro- and macroethical perspectives on engineering and technology in active and deliberative ways. We perceived it as compelling to examine the reflections articulated by engineering students, most of whom where in the final stages of their education, under the conditions of reflective freedom provided by the course design (see sections  “Course Syllabus and Design” and “Employing Pedagogical Flexibility”).

### The Case in Context

All students in the study were enrolled in an engineering program in Sweden, where such programs extend over five years and lead to both a professional degree and a Master’s of Science degree. There is no national standard for workplace-based learning that applies to all master’s programs in engineering. It is, however, nationally regulated that they should engage with ethics and societal perspectives on technology and engineering during their education. In relation to this requirement, the studied fiction-based EEE course was offered as one of several elective courses to students across all engineering disciplines at a Swedish university. Originally, the course had run on campus, but in the wake of the covid pandemic it was held online when data for this study was collected.

#### Course Syllabus and Design

The intended learning outcomes (ILOs) of the course were divided into two categories. The first category focused on generic professional skills such as communication, collaboration, and dealing with wicked problems. The second category related to students’ ability to reflect on ethical dimensions of technological development and to use fiction as a resource for such reflection. The ILOs of the second category required students to demonstrate awareness, articulate their positions, and create texts that guide others through complex ethical issues.

**Fig. 1:**
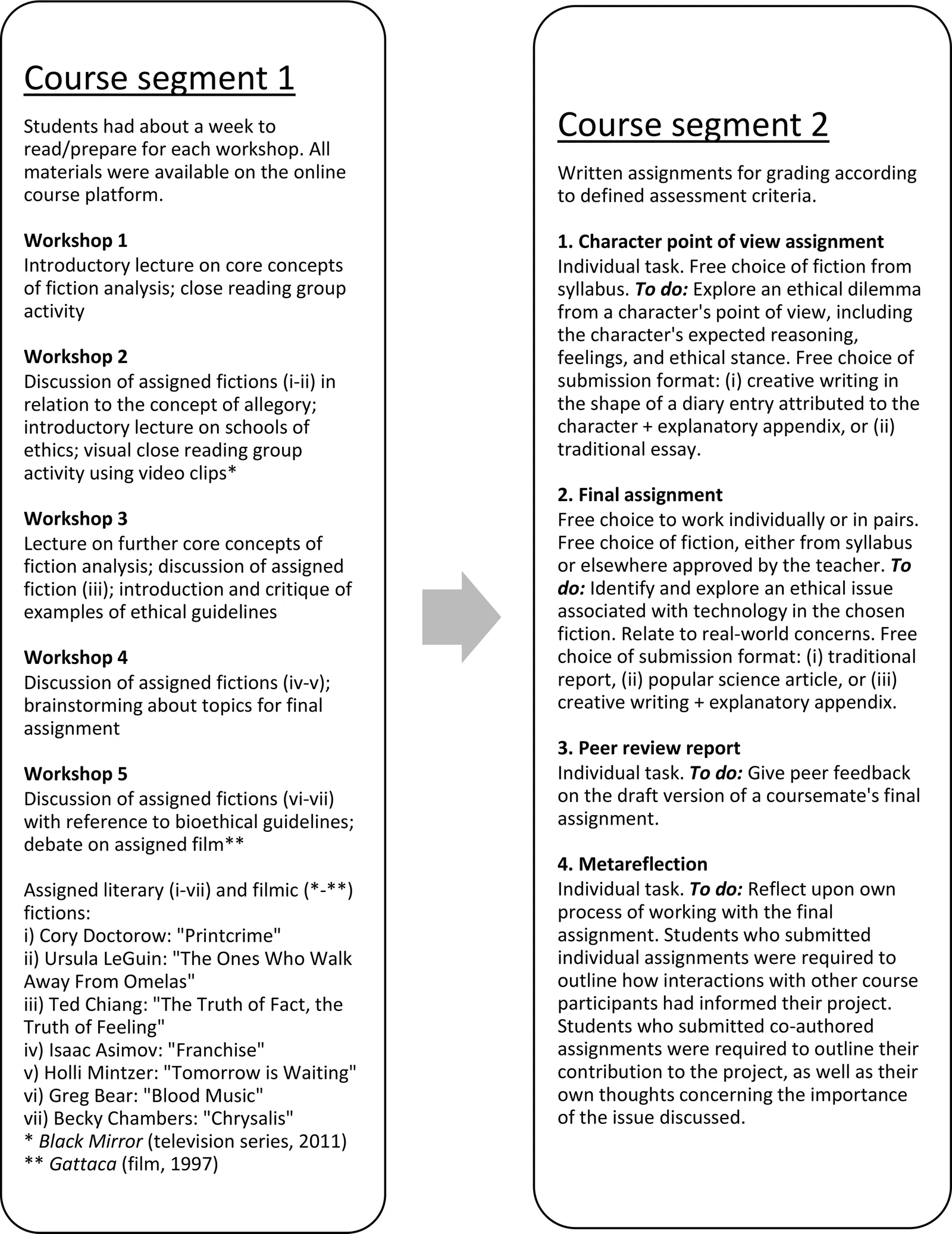
Course structure and content

The scope of the course was the equivalent of five weeks’ full-time study, distributed over a semester with a two-segment structure. The first segment comprised five weekly workshops centred around assigned fiction readings. Each workshop was facilitated by the same teacher, who introduced analytical frameworks, and moderated the student discussions. The second segment consisted of independent work with four course assignments. For a detailed description of the content and course design, see Fig. 1.

### Participants

Using purposive sampling (Merriam, 1998), the teacher was recruited via the first author’s academic network. The teacher had a background in literary studies and had worked in engineering education for several years, primarily teaching academic writing. When data was collected, the teacher taught the course for the third time.

The first author recruited students in the course introduction. From the outset, the course had 16 enrolled students and twelve of them consented to participate in the study. Because of dropouts from the course, the final sample consisted of nine students (Student 1‒9) from nine different engineering disciplines. Among these, six students completed the course, while the other three still generated some data for analysis.

The participating students were all in their twenties. One was participating in the course as an ungraded alumnus, who only joined for the workshops. The others had completed at least half of their program, and the majority were in their final year. Aside from the alumnus, the students did not have vocational experience as qualified engineers. However, some had had summer internships doing technical work at companies with a large body of engineering staff, and others had been introduced to professional research environments through their dissertation projects.

### Data Collection

Data triangulation was applied to study the case in its full complexity (Merriam, 1998; Yazan, 2015). Only qualitative data were collected, including workshop observations, students’ written assignments, and individual interviews with the teacher and four students who had completed their final assignment and consented to be interviewed. Also, the syllabus and all course materials were gathered for contextual understanding. For an overview of the students’ data contributions, see Table 1.

**Table 1:** Students’ data contributions

Student	Active in workshop discussions	Completion of written assignments (1–4)	Interview
1	Yes	All	Yes
2	No	All	Yes
3	Yes	All	Yes
4	Yes	All	Yes
5	No	All	No
6	No	All	No
7	No	Assignment 1	No
8	Yes	None	No
9	Yes	None	No

#### Workshop Observations

The first author prepared for observing all workshops by reading/viewing the assigned course fictions according to the syllabus. All workshops were initially observed in real time through attendance online and thereafter recordings of the sessions were revisited several times on the online platform of the course to further develop the fieldnotes. Sequences with non-study participants were only briefly commented in these notes and not included in the analysis.

#### Written Assignments

All written assignments of the course were collected, as far as they were completed by the students in our study. Also, all materials connected to the assignments were gathered including the teacher’s written and oral instructions, workshop slides and devices (e.g. discussion questions, analytical frameworks), and prescribed assessment criteria.

#### Individual Interviews

All interviews followed prepared interview guides along with follow-up questions based on the participant’s testimony. The interviews were recorded and transcribed verbatim for analysis, while quotes in the results have been translated from Swedish to English.

The teacher was interviewed once before the course started, and once again after its completion. Each interview lasted for about one hour, of which the first focused on the teacher’s pedagogical ambitions and the overall educational context of the course, and the last gathered the teacher’s thoughts on how the course connected to the engineering profession.

The four students, who engaged in this part of the data collection, were interviewed separately after the course for about 30 to 45 min. During the interviews, students shared their: Motivation and expectations before the course started; Experiences of the course fictions and activities; and Perceived relevance for their future engineering profession.

### Analysis

Data analysis progressed through the phases of consolidation, reduction, and interpretation (Merriam, 1998), as outlined below.

In the consolidation phase, initial analysis ran parallel with collecting all data and materials. Focused meanings were highlighted, and reflective notes were made. When data collection was completed, all content was read/viewed multiple times to grasp the full case.

In the reduction phase, concentrating measures were taken in accordance with the research aim. Student data were reduced by only selecting data relating to students’ ethical reflections for analysis. Initial analysis of the reduced set of student data subsequently informed and delimited our analysis of other types of data.

At this juncture, the literature on fiction-based EEE was revisited to create an analytical framework that would both fit data and keep focus on the research aim. Along with the notions of micro- and macroethics, this framework came to embrace the four pedagogical potentials as outlined in the introduction: (i) application of theoretical approaches, (ii) altering positions, (iii) consideration of context, and (iv) imagining possible futures.

In the interpretation phase, the reduced student data were sorted into five clusters: *micro-personal*, i.e. reflections about individual ethics based on personal values or experiences; *micro-professional*, i.e. reflections about the ethics of individual engineers in their professional context; *micro/macro*, i.e. reflections where one and the same student idea involved both ethical levels; *macro-professional*, i.e. reflections about the ethical responsibilities of the engineering profession as a collective; and *macro-societal*, i.e. reflections about ethical issues in the interplay between technology and society. The contents of these clusters were then considered in the light of the four pedagogical potentials of fiction. Through this process of clustering and projection, typical reflective patterns could be discerned. In the second tier of analysis, the identified reflective patterns were related to the teacher data (from workshops and interviews), fictions, course material, assignment instructions, assessment criteria, and student interviews.

### Researchers’ Positionality

Neither of the authors have a background in engineering, and neither of them were involved in the teaching or design of the course, which likely limited their understanding of the case. Rather, both were researchers within the field of higher education. The first author had a background in literary studies, and experience of facilitating fiction-based reflection and creative writing in professional education settings, which fed into the data collection and analyses. The second author had extensive experience of higher educational development, although not within fiction-based teaching. From this position, the second author provided critical distance to the case, especially in the analyses and joint writing process.

### Ethics

This study was approved by the Swedish Ethical Review Authority (ref. 2021-06193-01). All participants gave their informed consent to participate in the study, and they could withdraw from the project at any time. Also, an early report of the results was validated by the teacher, and interviewed students were invited to approve their own quotes included in this article.

## Findings

The studied course was given as part of an institutional initiative to support students’ development of professional skills, including ethical competencies. It is therefore noteworthy that the four interviewed students did not choose to take this elective course because they were concerned about acquiring professional skills in ethics in the first place. Rather, their common rationale was that they thought it would be a ‘fun’ course compared to the alternatives, either for the opportunity to engage in philosophical discussions on ethics in general (Student 1), or for the opportunity to read fictions (Students 2‒4). Similar reasons could partly explain the finding that students did not orient themselves towards discussing their own future profession when they engaged with the fictions. However, this remarkable result could also be related to other factors, such as the teacher’s pedagogy and the students’ previous professional experiences, as explicated below.

### The Teacher’s Pedagogy

#### Making Students ‘Round Themselves out’

All workshop discussions and written assignments were grounded in fictions: mainly the ones selected by the teacher, but also to some extent other fictions that students brought up. The notion that students through engagement with fiction could begin to ‘round themselves out’ was central to the teacher’s pedagogy, and the teacher thought that:

The fiction might get us to a point where we have feelings we don’t usually willingly experience, and that can be… good if it allows us to start thinking about why we feel that way […] I think it allows for some intellectual engagement on these emotional topics and as much as those engineers and scientists might think there’s no emotion in engineering or science, what they do has consequences on the lives of others and it’s important that they at least think a bit about that.

(Teacher, interview 2)

The teacher stated that the course fictions had been selected on the criteria that they should be ‘closely relating to something that would be in students’ professional life’ (Teacher, interview 2). Hence, all fictions depicted the development and/or impact of new technologies, except the short story “The ones who walk away from Omelas”. That story had still been included in the reading list by the teacher because of its ‘very heavy emphasis on ethicality in a kind of allegorical or metaphorical way’ so it would be ‘easy to relate to technology’ anyway (Teacher, interview 2). According to the student interviews, they had enjoyed the selected fictions, which Student 4 described as ‘classical sci-fi, although one noticed that they were handpicked to sharpen the reader’s mind, to actually think about problems.’

During the first course session, students were informed that the teacher had no intention of directly forming their ethical values, but rather to develop their ethical reflection habits and extend their awareness of and engagement with ethical issues regarding emerging technologies. It was further explained that the use of fiction was meant to provide a testing ground for ideas and an aid to sustain introspective thinking and reflection on how technical work might be made more humane (Teacher, workshop 1). In subsequent workshops the teacher also reminded students about the importance of self-reflection although they were not explicitly required to expose their personal viewpoints.

#### Employing Pedagogical Flexibility

The teacher viewed flexible course activities as a way of safeguarding the desired pedagogical function of fiction as ‘an unbounded field’ (Teacher, interview 2) in which students could experiment in their reflections, rather than be pushed towards preconceived ideas.

The flexibility of the course design had four mainstays. First, *reading questions and discussions prompts* offered flexibility by positioning a range of different approaches to the fictions as acceptable and relevant in course work, including close reading, perspective-taking, introspective, critical-philosophical, and engineering design approaches (see example questions in Table 2). In the workshops, the teacher would often refer to a range of prompts and let students choose what to discuss. Second, active *discussion participation* was encouraged but voluntary. This meant that although some of the reflection prompts offered by the teacher inquired after personal views, students were not required to articulate them. Third, while the *use of professional ethics guidelines* as analytical tools for defining ethical dilemmas in fictions was mandatory in the final assignment, students were encouraged to seek out guidelines that they themselves considered relevant to their course work. Finally, *assignments* were flexible in terms of both form and content.

**Table 2:** Types of prompts for discussion and reflection

Prompt type, by encouraged approach	Example, prompt provided to students as basis for workshop discussions
Close reading approach	‘What is the setting for this story? Whose point of view do readers experience? In a sentence or so, what do you think is/are the main theme(s) of the story?’ (provided for all literary fictions)
Perspective-taking approach	‘Vergil calls himself a “super-mother,” which Edward corrects to “super-host” … What do these two labels indicate about their speaker’s different points of view about Vergil’s experiment?’ (for “Blood Music”)
Introspective approach	‘If Remem [technology for recording everything a person sees and hears and storing it in a personal, searchable database] were real, would you want to use it? Why or why not?’ (for “The Truth of Fact, the Truth of Feeling”)
Critical-philosophical approach	‘Is equal access to technology an ethical goal? Are there some technologies that should not be available to everyone? If so, which ones, and how can we draw the lines of where to decide that?’ (for “Printcrime”)
Engineering design approach	‘Because so much of AI implementation relies on data, how (if at all) can creators of AI be sure that that their data choices are not only ethical, but realistic? And what is at risk if the data on which AI are based does not represent reality, but instead creates – or fictionalizes – it?’ (for “The Truth of Fact, the Truth of Feeling”)

For both the character point-of-view assignment and the final assignment, students were given a choice of format, with one creative writing option and one formal report option. While it was mandatory to focus on a fictive representation of a technology-related ethical issue in both assignments, students could choose what issue to explore and what literature (fiction, non-fiction, and ethics guidelines) to use in that exploration.

In the final assignment, students were additionally offered a third option of writing a popular science essay. Each final assignment option had unique assessment criteria, which to some extent emphasized different learning outcomes. For instance, only the popular science and creative writing options were aimed at practicing ‘communicating ethical-technological issues to a wider audience’ while the report option had a requirement that was absent in the other two: ‘your report should answer the question: What technology ethical issue does this text contain, and how might real-world creators of that technology incorporate ethical reflection on this issue into their work?’ (Instructions, final assignment). The overarching intended learning outcomes of the course were generic enough to encompass this variety.

### Student Reflections Within the Fictive Sphere

Within the fictive sphere, i.e. with sole reference to the world or events of a fiction, the written and oral work of the students clearly reflected that they were able to identify ethical problems in the fictions. They also realised the pedagogical potentials of applying different theoretical approaches (e.g. schools of ethics), empathetically altering their positions, and considering the context. In these regards, students typically focused on the microethical dimension, i.e. at the level of individual characters, although some of their reflections embraced the macroethical dimension of fictional worlds as well. The students’ focus on the behavior of characters had two salient consequences: firstly, that students *unvoiced self-reflection*, meaning that their reasoning regarding something impersonal – in this instance the thinking and actions of fictional characters – was articulated in the stead of reasoning about their own thinking and actions; and secondly, that in their engagement with the fictions, students *inhabited non-professional fictional worlds* since the characters were not placed in professional settings. These findings could partly be related to the teacher’s pedagogy, as outlined below.

#### Unvoicing Self-Reflection

In interview, the teacher mentioned that some topics could be sensitive to talk about for personal reasons. For instance, the selected movie *Gattaca* was one such example because it ‘deals with issues of genetic engineering’ which could be difficult for ‘someone who is very sensitive about disability’ (Teacher, interview 1). Therefore, the teacher had carefully chosen not to push any student to share their self-reflections and designed the assignments accordingly. For instance, in the ‘character point-of-view’ assignment students were instructed to go beyond what was already spelled out in an ethical dilemma of the fiction – but still keep their own values off point in their text:

The goal here is to think of an ethical dilemma, and of course when we think about those, we have to think about our own viewpoints, stances, and opinions, but we also have you having someone else’s ethics in mind as well […] In any ethical thinking, our own opinions are going to come up, but for what you write in this text, you will focus on their [the character’s] point of view.

(Teacher, workshop 2)

In their work with the assignment, students clearly showed their ability to empathetically alter position within the fictive sphere and to engage actively in ethical reasoning from that position of alterity. This is exemplified in the text of Student 2, who explained why the main character Edward decided to kill his microchip-invaded (human) friend Virgil in the end of the short story of “Blood Music”:

My main argument for why I think that Edward thinks that the ethical thing to do would be to get rid of the chips at any cost is that Edward kills Vergil in the end of the story… I don’t think he does it out of hatred because the friendship between Vergil and Edward is described as pretty genuine. They are not really close anymore and they have their differences, but they have shared a lot in the past and genuinely care for each other’s wellbeing. The main proof of this is that Vergil entrusts Edward to help him, and Edward truly tries. However, despite of this deep friendship the story ends with Edward killing Vergil. This really shows that Edward values the safety of the rest of the world over his friend’s life and the scientific advancement.

(Student 2, ‘point-of-view’ assignment)

Even when students weren’t explicitly instructed to *unvoice* their self-reflections as in the ‘character point-of-view’ assignment, their tendency to do so was palpable. A particularly clear articulation of a student’s preference to focus on the general rather than the personal could be found in the final assignment of Student 1:

Looking at the … works I have created or participated in, in this course, a clear theme emerges about the fear of giving up autonomy. As I prefer to consider general ideas and the big-picture, I think this tug of war between optimisation on the one hand and the private sense of control on the other is one the most critical problems for individuals in the near-term future.

(Student 1, final assignment)

In this study, it largely remains unknown to what extent students pondered their own viewpoints while doing the course assignments. However, in the interviews, students mentioned that the course fictions did make them think about ‘things one had not thought about before’ (Student 1 and 2).

#### Inhabiting Non-Professional Fictional Worlds

In some student analyses, the focal issue was clearly personal (to a character) rather than professional. For instance, Student 6 submitted an analysis of the ethical considerations of a character who, as a parent, had to decide whether to allow their child to be altered through biotechnological means. In other cases, the professional proximity was greater, but the situations under analysis were still not representations of professional engineering practice. An example of such a case was found in the creative writing of Student 3. Inspired by the short story of “Tomorrow is Waiting”, which revolves around a college student who successfully creates a sentient AI Muppet, Student 3 outlined a potential future in which advanced AI had become illegal because of numerous incidents in the past ’where developed algorithms behaved in unpredictable and destructive ways’. Still, the main character of Student 3’s short story – who was also a computer engineering student – created a sentient AI entity to win a competition with the following reasoning in mind:

The competition was lingering in the back of Alex’s mind as she went to her classes. Being a student, she wouldn’t mind getting a little extra money, and the internship would be a great opportunity as well … What she really wanted to do was a machine learning algorithm … The problem was that she wasn’t really allowed to develop machine learning algorithms yet, as she hadn’t finished the course about AI laws and ethics. She pretended to reflect on it but had already made up her mind: ‘If I’m really careful, and if I only use the most basic principles, it should be fine.’

(Student 3, final assignment)

In an appendix to the short story, Student 3 discussed ethical perspectives on the main character’s behavior with reference to an ethical framework regarding AI development issued by the European Commission. This discussion contained several points where the student realized the pedagogical potential of considering context as far as societal context goes, including that the main character broke the law, disregarded her own limited understanding, disregarded ‘other people’s concerns and the democratic decision of limiting the development of AI’, and put too few control-mechanisms in place. However, there was no connection made to professional context, e.g. hierarchies, productivity pressures, culture, or collaboration in the workplace, which is unsurprising given the fictional setting. Moreover, the interview responses of Student 3 revealed that they considered their future professional work as fundamentally different from what the course fictions depicted, as it would be contained within a larger, and compartmentalized, organizational framework. The main character of their short story, on the other hand, worked alone, developing new technology in an uninsulated situation.

### Student Reflections Beyond the Fictive Sphere

In their reflective transfer from fiction to reality, students tended to target the macro-societal dimension in the first place, where they realised the pedagogical potentials of applying theoretical approaches and imagining possible futures. Typically, students based their leaps beyond fiction on their everyday understanding, including theoretical frameworks acquired outside the course. In the written assignments, they also integrated ethical frameworks introduced by the teacher, such as utilitarianism and deontology. Distinctly micro-personal, micro-professional, or macro-professional perspectives were absent as students’ thus addressed a potential future in real life by *imagining possible consequences for society.* Sometimes they also connected to the present by *revealing critical opinions on society.*

#### Imagining Possible Consequences for Society

In their discussions, students commonly raised macroethical questions regarding the societal consequences if scenarios in the fictions were to come real. For example, in relation to the short story of “The Truth of Fact, the Truth of Feeling”, in which human memory is outsourced with the aid of technology and thus made infallible and searchable, Student 9 stated in workshop 3 that: ‘I think a major issue is who has access to these memories and how that effects how we dare act, since someone else might see it’. Although students’ leaps from fiction to reality could often be accompanied with a personal standpoint, such as ‘I think’, these positionings were not further elaborated on the micro-personal level, e.g. in terms of personal values or experiences. Neither did the teacher ask for such elaboration, which aligns with their deliberate decision not to push any student to expose themselves.

The same circumstance holds for the written assignments. An example of this type of impersonal reasoning is found in the writing of Student 1, who reflected upon the aforementioned fiction “Tomorrow is waiting”:

Due to anthropic bias, we often overlook the fact that new sentient beings need not look like us. When a muppet suddenly appears human-like, a cosmological horror, which is the tension of the story, emerges. Part of this [collective] fear is about the inconceivability of the nature of that being. Another part concerns the goal of the being. The orthogonality thesis states that an artificial intelligence can have any combination of intelligence level and goal. An increasingly complex paper-maximizing machine is not the stuff of horror movies but, uncomfortably enough, it could be a real threat.

(Student 1, ‘point-of-view’ assignment)

The use of a generic ’we’, exemplified by the quote above, was recurring in the students’ reflective writing regarding the possible societal consequences of emerging technologies, particularly AI. The students’ writing signaled that they included themselves in the ‘we’ on the grounds of being part of society and users of technology. They did not express that they considered themselves to be part of, or on their way to becoming part of, any particular subgroup within that larger collective.

#### Revealing Critical Opinions on Society

The pattern of *unvoicing* was not absolute when the students used fiction as a point of departure to consider ethical issues existing in real society. In such discussions, students’ personal and critical opinions did sometimes emerge. For instance, in workshop 4, the teacher asked the students ‘if the selection of data was sufficient to make the selection process’ that was illustrated in the fiction of “Franchise”. In this short story, students had read about how the supercomputer Multivac had replaced conventional elections for the presidency in the USA, and how the main character Norman had been selected to be the human representative to answer questions for the computer’s calibration. Responding to the teacher’s question, Student 8 revealed their personal mistrust to AI programmers in real life:

That’s like a classic problem with AI things. You know … there was this police thingy that went racist… Who selects the data that the AI system is going to use? … Can I trust those people? I generally don’t trust those people… That racist AI… ended up being like – ‘yeah, statistically, black people do more crime, therefore, they will do more crime’ … but back up a little bit… Could it be that the programmers were white dudes?

(Student 8, workshop 4)

The interviewed students thought that such open-ended discussions in the course had been highly rewarding. Since the fictions could be interpreted in multiple ways that validated different perspectives beyond ‘fact is fact’ (Student 2), this also gave the students opportunities to adopt a critical approach to technology which they otherwise felt was lacking in their engineering programme:

It was a nice contrast to what you read about in *Nature* or *New Technology*, or in stuff where they just put: ‘opportunities, opportunities, opportunities.’ There are many lecturers, and what you normally read about technology is like: ‘Wow, how many opportunities we have with these new things.’ So, it was a bit refreshening to receive… ‘but look how bad it can become’. That does not feel equally discussed. Those who hit the brakes are not as many as those who just try to continue the flow.

(Student 4, interview)

#### Non-Engagement with Professional Practice

While the students’ attention was repeatedly brought to the ethical responsibilities of engineers, they were never pressed to express their own thoughts on professional ethics in the workshop setting or in written assignments. Rather, the teacher suggested that:

it’s … a good habit to – when reading or viewing fictions – think about the big issues there and … reflect on your own position as regards them … I’m not going to make anybody talk about those kinds of very individual, personal views but it’s useful because that lets you know as someone who is an engineer … what your own viewpoints are and that might inform what you do or do not do when you’re working on different kinds of technologies or advancements.

(Teacher, workshop 5)

Hence, the setup of most activities did not require students to express how the fictions connected with their future professional practice. Instead, the students’ discussions and reflections tended to focus on the philosophical or policy dimensions of ethical issues. For instance, in workshop 5, students had viewed the filmic fiction *Gattaca*, which unfolds in a near future where the controlling elite society consists of genetically modified human beings produced in laboratories. Natural human beings, for their part, had become second-class citizens with no rights to the privileged life of the artificial humans. In connection with the film, the teacher invited students to debate by saying:

If you are in room one, you are going to represent an interview panel at a company. So you kind of have the more corporate take on this. You have to justify why the use of DNA screens, kind of like they had in *Gattaca* … is sufficient to determine whether a candidate is suitable and qualified for a job … But if you are in room number two, you’re going to defend the job applicant in this scenario, and … say why the interview panel should look beyond just the DNA … as an indication of future job performance.

(Teacher, workshop 5)

In the debate that followed, the student teams offered philosophical, macro-societal reasoning about the effects of people being judged by their genes and thus realized the pedagogical potentials of applying theoretical approaches and considering (societal) context. Interestingly, they also pointed out factors that might inform corporate policy, including resource management. However, this was done without reference to ethical implications for engineers who, individually and collectively, turn policy into practice.

Similar observations could be made in the final assignment, where students’ focus on socio-philosophical or policy-oriented macrolevel reasoning was repeated. For instance, Student 1 and Student 5 executed the task by exploring the possible impact of extended dependency on AI on human society and autonomy, but did not draw explicit conclusions regarding how such considerations might manifest in the work of engineers, while Student 2 discussed how governments might address issues of transparency in AI-based public decision-making from a policy perspective rather than an engineering perspective.

#### Not my Role as an Engineer

Whereas the social responsibility of engineers was sometimes mentioned by the teacher, students tended not to use the words ‘engineer’ or ‘engineering’. In workshops and written assignments alike, they rather spoke more generically of ‘scientists’, ‘creators’ of technology, or ‘everyone who makes a specific tech possible’. It turned out in the student interviews that the absent engineering profession was not merely due to the profession’s absence in the fictions or to the keying of the course’s learning activities. According to the interviewed students’ prior experiences and career expectancies, engineers focus on delimited technical tasks and therefore have no ethical agency in practice. Student 1 explained that:

There’s not much focus [on ethics in professional practice] at all. There are specific positions for people who are meant to engage with these issues, and they aren’t engineers… When I worked [as in intern in industry] … I had nothing to do with it [ethics]. I only had my specific tasks and was supposed to focus on them.

(Student 1, interview)

Student 3 instead trusted the process, and perceived their future professional role as insulated from real-world damage:

For my part it [the course] didn’t feel that relevant. The way things look now, I want to work in plasma physics and… like, do research, which means run simulations on my computer. And there are… there aren’t that many things that can go wrong there.

(Student 3, interview)

However, Student 4, who had chosen a fiction that aligned with their own engineering discipline as basis for the two major written assignments and was involved in a research project where they were going to ‘genetically modify a bunch of things’, was a bit more concerned:

“Blood Music” was genetic modification on a very advanced level. But as I wrote in one of my papers we’re kind of doing this, genetically modifying immune cells to treat cancer and so on. And then “Blood Music” has taken it a bit further, but it’s not a huge stretch. It’s like not very far from reality.

(Student 4, interview)

### Picturing the Pedagogical Relationships

**Fig. 2:**
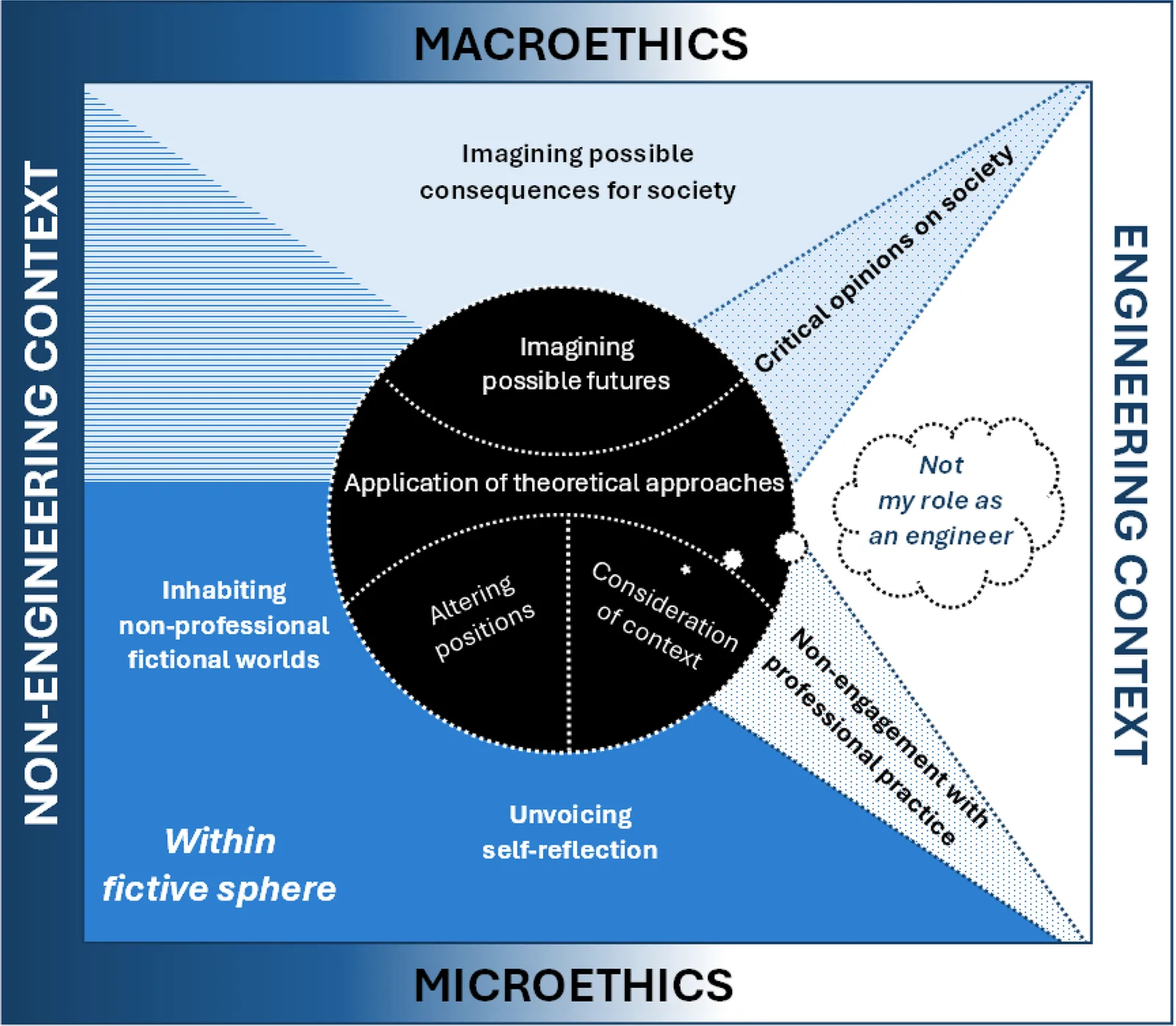
Students’ reflective engagement with fiction

The findings of this study can be understood as an account of how four pedagogical potentials of fiction-based EEE, identified through review of previous research (see section  “Four Pedagogical Potentials of Fiction-Based EEE”), were realized by students within the frame supplied by the course. As illustrated in Fig. 2, all the four potentials were realized in the students’ course work; however, they were realized in ways that did not connect explicitly with the students’ own future professional practice.

The figure shows how the students’ ethical reflections were framed within a continuum of two dimensions: Non-engineering vs. engineering contexts, and Macroethics vs. microethics. The non-engineering pole (dark blue) represents contexts where no one acts or identifies as an engineer, while the engineering pole (white) includes contexts that are characterized by professional engineering practice. The continuum between these two poles permeates the vertical dimension as both macroethics and microethics could be more or less oriented to the engineering practice. Moreover, students’ reflections could be more or less oriented towards macroethics (upper frame) or microethics (lower frame).

The four pedagogical potentials of fiction-based EEE are centered in the black core, which is connected to students’ reflective activities (surrounding fields in blue/white). With respect to students’ thought that engineering ethics is ‘not my role as an engineer’ (thought bubble), it reflects no realization of the four pedagogical potentials of using fiction in EEE. However, students *did* consider their knowledge and expectations regarding professional engineering contexts when coming up with this conclusion (see string of thought bubbles).

## Discussion

This study shows how engineering students, within a fiction-based EEE course, used fiction as a reflective device to engage with ethical issues connected to the use and development of technology; how their engagement realized pedagogical potentials of fiction-based EEE; and how the findings could be understood in relation to the pedagogical framing and activities.

Even though the students realized all the four predefined pedagogical potentials of fiction-based EEE (application of theoretical approaches, altering of positions, consideration of contextual factors, and imagination of possible futures), findings show that they seldom connected their ethical reflections to engineering practice and even less to the macro-ethical responsibilities of the engineering profession or their own, personal ethics as future engineers. We view these findings in relation to the circumstance that students were not required to explicitly make those connections. Discussion prompts and assignments offered opportunities to either address ethics in professional engineering directly, or to leave it unaddressed. Interestingly, the two students who made the clearest connections between fiction-based insights and professional practice both chose to discuss a topic that aligned with their own discipline.

Relation of the study’s findings to extant scholarship sheds some light on factors that may have contributed to this dynamic, as outlined below.

### Unlocated Locus of Engineering Deliberation

The students’ tendency not to connect their ethical reflections to engineering practice can be understood in different ways. It is probable that the course being taught solely by a teacher from the humanities mattered in this regard, as it can be difficult for non-engineer faculty to frame their teaching in ways that facilitate such connections (Hansen, 2018; York, 2018). Moreover, student interviews indicated that the approach to technology and engineering ethics facilitated by the fiction itself may also have been significant. Typically, the interviewed students perceived the work of engineers as having the technical squarely in the foreground, with everything else receding into the background or occurring outside of the professional sphere – whereas fictions like “Printcrime”, “The Truth of Fact, the Truth of Feeling”, “Franchise”, “Tomorrow is Waiting”, “Blood Music”, and “Chrysalis” operate on a reversed logic: the technical is in the background, typically as a necessary but largely uninterrogated condition for the plot, while relationships, values, and emotions take centre stage.

Rather than seeing this reversal of emphasis as an aid towards becoming ‘whole’ engineers (Hitt et al., 2023), most interviewed students problematized how applicable insights from the fiction-based course were to their imminent professional practice (not very, they told the interviewer). However, they endorsed that reading and reflecting on fiction could widen their outlook and make them more deliberative as people, technology users, and citizens. It conforms with this point of view that these students preferred to apply philosophical, societal, or policy perspectives in their coursework since that is where they saw loci for deliberation; however, the question of why they did not see a corresponding locus in engineering practice remains.

The notion of engineering as purely technical-rational that becomes visible here pinpoints how a deliberative approach to professional practice is dependent on the ability to zoom out from tasks themselves to the bigger picture in which they are ethically situated. In engineering, this is a complex enterprise involving reconciliation of multiple accountabilities – to the interests of one’s employer, clients, or other stakeholders; to society, profession, and public welfare; and to personal morals (Smith, 2021). Deliberation in engineering is anchored in consideration of the human purpose (Blockley, 2015) of any scheme that one’s expertise is harnessed to promote. However, as engineers relate such considerations to practice, they must also deal with the distributed nature of agency in their profession – i.e., acting for and through others (Smith, 2021).

In our study, attention to the human purposes of technologies – on the micro and macro level, and beneficent as well as sinister – was something that the utilized approach to fiction-based reflection seemed to invite and support. However, the link between purpose and task that can lead to the deliberative locus outlined by Smith (2021) was not within the scope of the studied course, which may be significant in relation to the students’ sense of limited professional applicability. Notably, some scholarship exists that outlines possible approaches for introducing the task-purpose connection in fiction-based reflection, e.g. through integration of fiction-based EEE into technical courses (Odle & Bassett, 2021; Summet & Bates, 2020), interdisciplinary teaching and disciplinary consultants in curriculum design (Hansen, 2018, 2021), and reflective tasks that explicitly require students to formulate how they might apply fiction-based insights in their future profession (Sleezer & Bates, 2020).

### Unbridged Divides Between Fiction and Professional Practice

Students in our study became immersed in the fictions by discussing characters’ points of view in nuanced ways, which corresponds well with expectations on fiction-based EEE (e.g. Hitt & Lennerfors, 2022; Summet & Bates, 2020). However, the selected fictions generally portrayed scientists/creators of technology as independent agents, unsupported and unchecked by organizational structures. Thus, the dynamics of engineers’ professional environments (Bucciarelli, 2008; Conlon, 2022; McAninch, 2023) was missing in the fictions, and findings from the student interviews suggest that this may have limited the students’ tendency to connect the stories to their own future reality. Conversely, disciplinary alignment seemed to help students bridge the divide between fiction and practice to some extent. However, in the few instances where students used disciplinary knowledge to frame ethical problems in fictions as issues of engineering practice, they did so from an impersonal, regulatory perspective that was focused on the need for fail-safes in the technical process rather than on their future selves – and other engineers – as particularly situated actors (compare Smith, 2021).

It can be theorized that the students in our study might have connected to engineering practice in more self-reflective ways if the simulations of social worlds (Mar & Oatley, 2008) provided by course readings had come closer to their understandings of the world of professional engineering. However, our findings provide no evidence to that effect. Moreover, finding such fictions is no easy task (Hitt & Lennerfors, 2022), particularly as engineers form a comparatively incoherent professional collective, working as they do in diverse functional areas, positions, and organization types (Nilsson, 2010). Students’ visions of their professional futures might consequently be either too vague or too narrowly focused on one known type of position or organization to make it feasible for teachers to find matching fictions.

It is also noteworthy that a one-sided focus on alignment disregards the potential of fiction to create reflective distance to accustomed or expected positions (Hitt & Lennerfors, 2022; Sleezer & Bates, 2020). Most students in our study were in the final year of their engineering program and those who were interviewed appeared able to associate the fictions with professional contexts – or rather, to articulate the disconnection(s) between them – if explicitly asked to do so. This speaks to the possibility of bridging the divide between fiction and practice not only through maximizing congruence, but through engaging critically with incongruence as well.

### Unvoicing of Students’ Previous Experiences

Student interviews revealed that some students perceived the idea of engineers’ social responsibility as a fiction per se in relation to their previous experiences of professional practice from e.g. summer internships. While some of them were critical to this perceived situation, they still thought they were just expected to be the enablers of technology while ‘others’ took responsibility for the social consequences, either within the organization (e.g. ethics officers) or in the surrounding society. Thus, the interviews showed that at least parts of the student group were able to verbalize a sense of ethical detachment that has been found among engineering students in previous research as well (Lönngren, 2021; Smith et al., 2021), and which presents a fundamental problem for EEE educators (Conlon, 2022; Smith et al., 2021; Xavier et al., 2022).

The circumstance that students were not required to engage explicitly with their perceptions of professional relevance in most learning activities likely contributed to these ideas being *unvoiced* in the classroom. If they had been articulated, the way they were in the interviews, it might have provided an opening for a critical discussion of what ethical deliberation in engineering is, where and how it happens, who is involved, and in what capacity. Consequently, it seems vital to prompt students to relate their professional experiences and/or expectations to their fiction-based reflections. The teacher could, for instance, ask students what kind of exemplars characters set (Hansen, 2021), what scope they see for emulating such exemplars in real-world engineering practice, what conditions might best support ethical agency and social responsibility in engineering (Conlon, 2022) – and crucially: what all this could concretely mean for them in their own future practice.

### Unsafe Space Avoided

In our case, the teacher emphasized how fiction could evoke important self-reflections about being part of the engineering profession. However, out of respect for their integrity and comfort, students were not required to disclose introspective thoughts in their course work. In this way, a perceived risk that the classroom might become an unsafe space was avoided; but under such conditions of avoidance, the possibility to enact the kind of pedagogy outlined above is limited.

Since personal ethical standpoints could indeed be sensitive for students to share with others, it is vital to create a ‘safe space’ for discussing discomfortable topics (Harless, 2018). In such a space, students are not supposed to be safe from feeling potential negative emotions associated with the topic of concern, but they should both feel and be safe from being judged by their teacher and peers. While pedagogy that demands explicit self-reflection has been used in fiction-based EEE (e.g. Jalali et al., 2021; Manià et al., 2018; Sleezer & Bates, 2020), it appears that the overall program design in our case, and the repercussions of the pandemic, created challenges in this regard. Certainly, interdisciplinary communication can facilitate critical dialogue from various angles in EEE (Hansen, 2021) but building the essential trust for a space to be safe (Harless, 2018) may be temporally difficult to achieve within just five workshops among students who do not know each other from before, especially in an online format.

### Limitations of the Study

It should be noted that our results do not capture the full picture of the case. Firstly, because the case was documented, described, and interpreted with specificity of focus. Secondly, not all students participated in the study, and among those who did, only six completed the course, and only four volunteered to be interviewed. The sample of interviewed students may be skewed towards students who were particularly engaged by the course, and therefore more disposed to discuss it with the researcher. Also, the course ran online. According to the teacher, discussions were somewhat less engaged in online iterations of the course, at least initially.

Still, the results from our case may be useful to understand similar aspects of fiction-based pedagogy in other contexts and guide teachers in making informed decisions about using narrative art in EEE. The transferability in this regard needs to be assessed by the reader, who can deduce to what extent the described situations correspond with those in another case (Polit & Beck, 2010).

## Conclusion

Even though the engineering students in this study reflected on both micro- and macroethical perspectives and realized several pedagogical potentials that are commonly associated with fiction-based EEE, they did not articulate fiction-based self-reflections in relation to their future engineering profession. Several factors likely played a part in this. Firstly, students did not explicitly connect fictions with professional practice unless required to do so, and most learning activities featured no such requirement. Secondly, students were allowed or instructed to unvoice self-reflections since the teacher judged this approach to be situationally appropriate. Thirdly, individual interviews showed that students struggled to connect fictions with their expectations of professional practice, but since they did not articulate this in class, learning activities could not engage with the perceived disconnects. Against this background, we posit that teachers need to explicitly ask students to respond to fiction by putting the experiences and expectations of professional practice that it evokes into words; in short, by telling their own stories.

### Future Research

The effects of engaging explicitly with students’ pre-existing expectations on social responsibility and ethical agency in engineering, and of using fictions that reflect professional social worlds, constitute interesting avenues to pursue in future research. Likewise, EEE teachers’ opportunities and potential strategies to create a safe space for learning deserves further attention – and in this regard, we suggest that the outcomes from online versus campus formats should be compared.

## Data Availability

Data is not available on account of confidentiality.
